# Nutrient transporters in broiler chickens: intestinal gene expression profiles, functional roles, and influencing factors

**DOI:** 10.1186/s40104-025-01302-w

**Published:** 2025-12-04

**Authors:** Vahideh Shay Sadr, Jose A. Quinteros, Sonia Yun Liu, Reza Barekatain

**Affiliations:** 1 School of Life and Environmental Sciences, Faculty of Science, The University of Sydney, Sydney, NSW 2006 Australia; 2 Poultry Research Foundation, The University of Sydney, Camden, NSW 2570 Australia; 3 Sydney School of Veterinary Science, Faculty of Science, The University of Sydney, Sydney, NSW 2006 Australia

**Keywords:** Broiler chickens, Gene expression, Growth performance, Nutrient homeostasis, Nutrient transporters

## Abstract

The primary role of the gastrointestinal tract in broiler chickens is nutrient assimilation, with transporter proteins facilitating the uptake of amino acids, peptides, monosaccharides, fatty acids, and minerals across the intestinal epithelium. Among these nutrient transporters, members of the solute carrier family are particularly important, and gene expression analyses targeting these transporters have provided informative insights into how birds adapt to diverse dietary, environmental, and physiological challenges to maintain nutrient homeostasis. These transporters are expressed either at the brush border membrane, where they facilitate the absorption of nutrients from the gut lumen into enterocytes, or at the basolateral membrane, where they mediate the transfer of nutrients from the enterocytes into the bloodstream. The expression of these transporters is influenced by a range of factors, including bird age, sex, intestinal segment, dietary substrate availability and source, as well as external stressors such as heat stress and pathogen exposure. While upregulation of transporter genes often suggests an enhanced capacity for nutrient uptake, it does not always correlate with improved growth performance, due to compensatory physiological responses and fluctuations in nutrient bioavailability. Understanding the regulation and functional dynamics of nutrient transporters presents valuable opportunities to develop targeted dietary and management strategies aimed at optimizing nutrient utilization and improving bird performance. This review summarizes current knowledge on the classification, function, and regulation of key nutrient transporters in broilers, highlights factors influencing their expression, and explores their implications for nutrition and production efficiency.

## Introduction

The significance of poultry in human nutrition is irrefutable, as chicken meat and eggs constitute the largest sources of high-quality animal protein globally. Chicken meat is particularly appreciated for its affordability, low fat content, and absence of religious restrictions, making it a universally accepted and accessible protein source [1]. This growing demand for poultry products has fuelled extensive research in poultry nutrition, focusing on enhancing feed efficiency, welfare, and the production of premium-quality products. In modern poultry nutrition research, host gene expression analysis has become a powerful tool for investigating how birds respond at the molecular level to various factors such as dietary interventions, environmental stressors, and pathogen exposure. This process involves measuring changes in mRNA levels, indicating which genes are upregulated (increased expression) or downregulated (decreased expression) in response to various interventions. This enhances our understanding of the underlying mechanisms associated with changes in growth performance and nutrient utilization [2]. The 1990s marked the advent of gene expression studies in broilers, focusing on metabolism-related genes [3, 4]. By the early 2000s, the field had advanced significantly, emphasizing the role of specific dietary components in modulating gene expression.

The nutrient transporter genes are the most studied genes in poultry nutrition research. These transporters play a significant role in nutrient utilization, regulating the absorption of different nutrients such as carbohydrates, amino acids, peptides, fatty acids, and minerals. For instance, Gilbert et al. [5] demonstrated that dietary protein quality influences the expression of nutrient transporter genes, highlighting the direct impact of diet composition on nutrient absorption and metabolism. The expression of nutrient transporter genes varies across different segments of the GIT, reflecting their specialized roles in nutrient absorption. For example, sodium-glucose co-transporter 1 (SGLT1) is predominantly expressed in the duodenum and jejunum, where it facilitates glucose uptake. Similarly, peptide transporter 1 (PepT1) is highly expressed in the proximal regions of small intestine, allowing the uptake of di- and tri-peptides, whereas amino acid transporters such as excitatory amino acid transporter 3 (EAAT3) and L-type amino acid transporter 1 (LAT1) regulate amino acid absorption across the intestinal epithelium. Several factors can affect the level of expression of single or multiple nutrient transporters genes. These factors include, but are not limited to, dietary factors such as fibre source and particle size [6], amino acids [7, 8], protein quality [5] and levels [9], as well as environmental factors like ambient temperature [10, 11], and pathogen exposure [12]. Apart from these external factors, bird related factors specially age [13] and sex [14] or the interaction between age and sex [15] significantly impact gene expression of nutrient transporters.

While changes in gene expression can influence nutrient uptake, growth outcomes are determined by a complex interplay of factors including compensatory regulating mechanisms [16], nutrient availability, metabolic efficiency, energy costs [17], post-transcriptional modifications [18, 19], and environmental conditions [10]. As a result, increased nutrient transporter expression does not necessarily translate to enhanced nutrient assimilation or utilization, just as decreased expression does not always indicate impaired growth. This paper was prepared given the lack of a recent review and the importance of understanding of nutrient transporters in poultry nutrition research. The first section provides a comprehensive overview and classification of the major nutrient transporters studied in poultry nutrition, highlighting their roles in the absorption of key nutrients across different sections of the gastrointestinal tract. The second section explores the external and internal factors that influence the expression levels of these transporters. We also further discuss the relationship between gene expression of nutrient transporters with growth factors and explain why the upregulation or downregulation of nutrient transporter genes does not always correlate directly with improved or diminished growth performance.

## Nutrient transporters: classification and function

Feed conversion efficiency may be optimized by balanced digestive dynamics of energy and protein, which includes digestion, absorption and metabolism of nutrients [20]. At hatch, chicks have an underdeveloped digestive system, and pancreatic enzyme activity increases only gradually with age. As a result, the digestion of macronutrients in typical broiler diets may be limited during the early post-hatch period. Specifically, maximal amylase and lipase activities are reached by d 8, while maximal trypsin and chymotrypsin activities are attained by d 11 post-hatch [21]. Consequently, the absorption of nutrients is likely the key rate-limiting factor for the bioavailability of amino acids and glucose in modern fast-growing broiler chickens [22]. Ferraris and Diamond [23] proposed that the regulation of nutrient transporters is an adaptive mechanism that aligns nutrient uptake capacity with the organism’s physiological requirements. Changes in nutrient transporter gene expression serve as indicators of altered nutrient uptake rates in response to host metabolic demands or external environmental conditions [24]. Such insights are critical for understanding how nutrient uptake efficiency can be modulated in response to dietary composition, developmental stages, or stressors.

Nutrient transporters, which belong to the Solute Carrier (SLC) family, are categorised as either brush border or basolateral membrane transporters. Their mRNA expression levels have been analyzed across various intestinal segments, including the duodenum, jejunum, ileum, and cecum, in poultry. These include amino acids, carbohydrates, fatty acids, minerals and complexed nutrient transporters which will be classified and discussed with more details later in this review paper.

### Amino acid transporters

Amino acids can be absorbed as small peptides or free amino acids, and most amino acids are absorbed as oligopeptides [25] and their intestinal uptakes via peptide transporter 1 (PepT1) encoded by *SLC15A1* are both rapid and energetically efficient [26]. In humans, when glycine was administered as a dipeptide, 30-min post-prandial glycine plasma levels were 94.7% higher (4.40 vs. 2.26 mg/100 mL) compared to monomeric glycine [27]. The absorption of oligopeptides is more efficient than that of monomeric amino acids, which leads to a significant disadvantage of low-protein diets where large quantity of synthetic or crystalline amino acids are included to replace soybean meal in broiler diets [28].

PepT1 functions both as a peptide transporter and also peptide sensor, therefore known as a “transceptor” [29, 30]. PepT1 facilitates the uptake of di- and tripeptides through a proton-dependent mechanism linked to the Na^+^/H^+^ exchanger and the Na^+^-K^+^ ATPase [29]. It can transport most of the dipeptides and tripeptides but does not accommodate free amino acids, tetrapeptides, or larger peptides [24]. Peptide transporters are considered fundamentally significant in the process of cell survival and their gene expression can be upregulated under inflammation or stress conditions [9, 31]. Hence, the expression of PepT1 can also be regarded as a potential gut health biomarker in poultry.

**Fig. 1:**
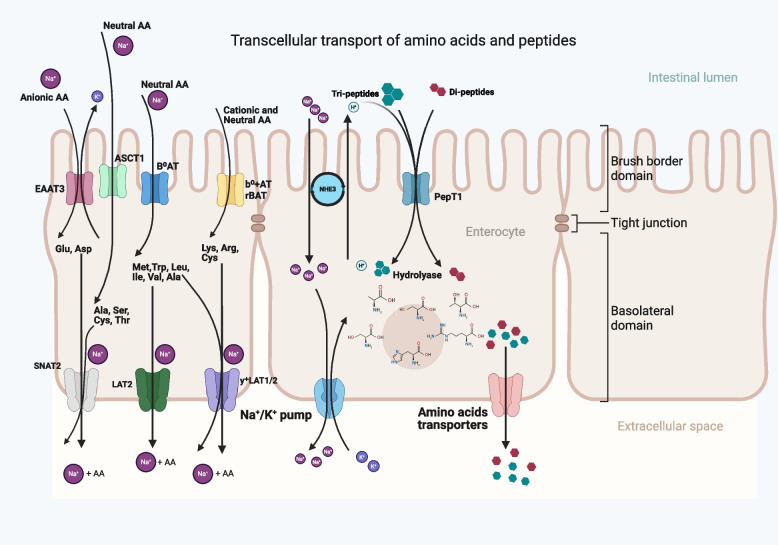
Transcellular transport of amino acids and peptides across the intestinal epithelium in poultry (created with BioRender.com). This figure illustrates the coordinated uptake and intracellular transport of amino acids and peptides in the small intestine. On the apical (brush border) membrane of enterocytes, a range of sodium-dependent and -independent transporters mediate the selective absorption of amino acids. Anionic amino acids such as glutamate and aspartate are transported via EAAT3, while ASCT1 transport small neutral amino acids including alanine, serine, cysteine, and threonine. B^0^AT facilitates the uptake of other neutral amino acids, such as methionine, tryptophan, leucine, isoleucine, valine, and alanine. Cationic and some neutral amino acids, including lysine, arginine, and cysteine, are taken up through heteromeric transporters like b^0^^,^^+^AT and rBAT. Peptides are absorbed via the proton-coupled transporter PepT1, which transports both di- and tri-peptides into the enterocyte. Cytosolic peptidases hydrolyze the absorbed peptides into free amino acids that are then available for transport across the basolateral membrane into the bloodstream. The Na^+^/H^+^ exchanger 3 (NHE3) helps maintain the proton gradient required for PepT1 function. On the basolateral membrane, absorbed amino acids are exported into the bloodstream through specific amino acid transporters such as SNAT2, LAT2 and y^+^LAT1/2 ensuring delivery to peripheral tissues. The basolateral Na^+^/K^+^-ATPase maintains electrochemical gradients required for amino acid transport across both membranes

The transport of free amino acids is mediated by members of the SLC family, with specificity for anionic, cationic, or neutral amino acids [32]. As illustrated in Fig. 1, the *SLC1A1* family of transporters includes the excitatory amino acid transporter (EAAT3), which facilitates the transport of anionic amino acids like glutamate and aspartate across the brush border membrane, and Alanine, Serine, Cysteine-preferring transporter 1 (ASCT1), responsible for transporting neutral amino acids such as alanine, serine, cysteine, and threonine at the basolateral membrane [33]. The EAAT3 is especially important as it transports glutamate, the primary energy source for intestinal epithelial cells. The gut expresses several other transporters. The list include sodium-independent cationic amino acid transporters, CAT1 and CAT2, part of the *SLC7A1* family [34], the L-type amino acid transporter 1 (LAT1; *SLC7A5*), system y^+^L-type amino acid transporters (y^+^LAT1; *SLC7A7* and y^+^LAT2; *SLC7A6*) [35] and the sodium-coupled neutral amino acid transporter (SNAT2; *SLC38A2*) [36]. Additionally, the heteromeric transporters rBAT (*SLC3A1*) and b^0^^,^^+^AT (*SLC7A9*), and the neutral amino acid transporters ATB^0^^+^ (*SLC6A14*) and B^0^AT (*SLC6A19*) also play critical roles in amino acid absorption and homeostasis [37, 38]. B^0^AT facilitates methionine uptake, while rBAT and b^0^^,^^+^AT are involved in uptake of lysine, arginine and cysteine [39], and ATB^0^^+^ is responsible for the uptake of leucine and phenylalanine [35].

### Carbohydrates transporters

**Fig. 2:**
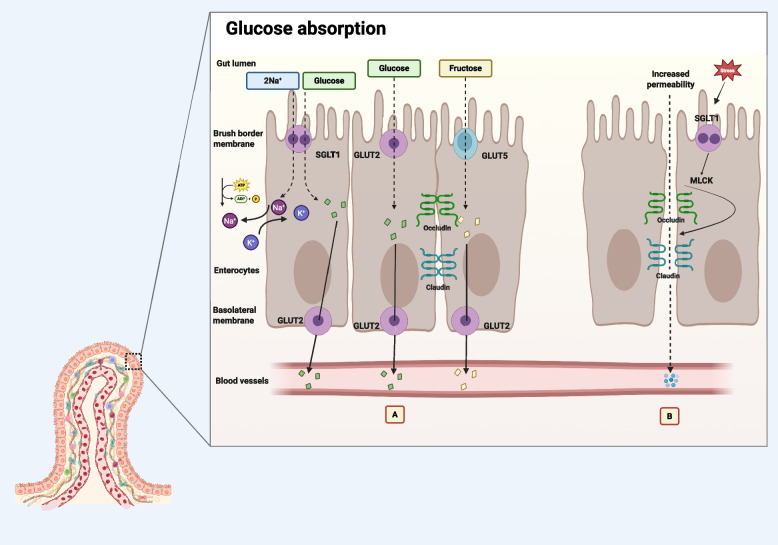
Overview of monosaccharide absorption pathways across the intestinal epithelium in poultry (created with BioRender.com). **A** Glucose and sodium (Na^+^) are co-transported from the gut lumen into enterocytes via the sodium-glucose co-transporter 1 (SGLT1) located on the apical (brush border) membrane. This active transport relies on the sodium gradient maintained by the Na^+^/K^+^-ATPase pump on the basolateral membrane, which exports Na^+^ out of the cell in exchange for K^+^. Once inside the enterocyte, glucose is transported across the basolateral membrane into the bloodstream by facilitated diffusion through glucose transporter 2 (GLUT2). Fructose is absorbed independently through the apical GLUT5 transporter and exits through basolateral GLUT2 in a similar manner. **B** Overactivation of SGLT1 may result in increased intestinal permeability through stimulation of myosin light chain kinase (MLCK) activity, leading to phosphorylation of myosin light chains (MLC), which promotes actomyosin ring contraction adjacent to tight junctions

Monosaccharide transport into or out of enterocytes is mediated by key transporters such as sodium-dependent glucose transporter 1 and 4 (SGLT1; *SLC5A1* and SGLT4; *SLC5A9*), and glucose transporter 2 and 5 (GLUT2; *SLC2A2* and GLUT5; *SLC2A5*). Glucose absorption in the gut primarily occurs via SGLT1 and facilitated diffusion through GLUT2, with paracellular absorption potentially contributing under certain conditions, which will be discussed later. At the brush border membrane, SGLT1 is the primary transporter responsible for glucose uptake, utilizing a sodium-dependent mechanism. SGLT4 also plays a role in transporting glucose, fructose, and mannose [40]. Under normal conditions, GLUT2 is primarily localized at the basolateral membrane, where it facilitates the transport of glucose, galactose, fructose, and mannose into the bloodstream. However, under high glucose demand, GLUT2 can relocate to the brush border, increasing monosaccharide absorption efficiency. In contrast, GLUT5 is exclusively responsible for fructose absorption and remains localized to the brush border membrane [41]. Evidence of paracellular glucose absorption has been documented in various mammals, including mice, rats, and rabbits [42], and in humans [43]. This form of absorption tends to become more prominent when the intestinal environment is altered, for example, during periods of elevated luminal glucose, compromised barrier function, or increased permeability due to stress or disease, allowing glucose to diffuse between intestinal cells. Karasov [44] speculates that birds and bats possess more permeable intestinal epithelia than non-flying mammals, enhancing their reliance on passive, paracellular nutrient uptake. These flying animals also have relatively short intestines and reduced surface area, an adaptation thought to lower the weight of the GIT and the digesta it carries [45, 46]. To offset this anatomical constraint and to compensate for any reduction in transporter-mediated absorption, they appear to rely more on passive absorption mechanisms to maintain adequate nutrient uptake. Although this phenomenon has not been explicitly studied in domesticated poultry, the physiological similarities and adaptive traits observed in birds suggest that broiler chickens may also utilize paracellular glucose absorption, especially under circumstances involving gut stress, elevated glucose requirements, or high luminal glucose concentrations. There is possibility of association between activity of SGLT1, myosin light chain kinase (MLCK) activation, MLC phosphorylation, tight junction contraction, and increased intestinal permeability [47] but this area of research is largely untapped in poultry, and more research is warranted to clarify association between glucose absorption and functionality of the intestine (Fig. 2).

### Fatty acid transporters

Fatty acid absorption in poultry occurs primarily in the small intestine, particularly in the duodenum and jejunum, with some absorption extending into the ileum. The efficiency of absorption depends on the type of fatty acid, its chain length, and whether it is in free form or part of triglycerides [48]. Short chain fatty acids (SCFAs) can cross the apical membrane of enterocytes primarily via passive diffusion and are subsequently absorbed into the mesenteric venous blood, entering the portal circulation [49]. In addition, the proton-coupled monocarboxylate transporter 1 (MCT1; *SLC16A1*) and the sodium-coupled monocarboxylate transporter 1 (SMCT1; *SLC5A8*) have also been identified to facilitate the uptake of SCFAs into intestinal epithelial cells both in mammals [50, 51], and broiler chickens [52]. Long chain fatty acids (LCFAs) absorption involves key transporters such as fatty acid translocase (CD36), fatty acid transport protein 4 (FATP4), and fatty acids binding proteins (FABPs) [53]. CD36, encoded by the *CD36* gene, plays a pivotal role in the uptake of LCFAs, facilitating their movement across the cell membrane for metabolic processes [54]. Similarly, FATP4, encoded by the *SLC27A4* gene, not only aids in fatty acid uptake but also links these molecules to intracellular metabolism, ensuring efficient utilization and storage [55].

In poultry, several FABP isoforms have been identified, each playing distinct roles in different tissues during the growth, reproduction and fat deposition [53]. FABP1 (L-FABP) is highly expressed in the liver of chickens, with significantly elevated levels during laying periods, suggesting its role in lipid mobilization for egg production [56]. FABP2 (I-FABP), found in enterocytes from the duodenum to ileum, facilitates dietary fatty acid absorption and intracellular trafficking and may protect cells from unesterified fatty acids toxicity [57, 58]. FABP3 (H-FABP) is expressed in the heart, skeletal muscle, and adipose tissues, and its expression correlates with abdominal fat deposition and peak egg production, making it a useful marker for fat related traits [56, 59]. FABP4 (A-FABP) is prominent in adipocytes, where its over expression enhances lipid accumulation and intramuscular fat deposition in chicken [60]. FABP5 (E-FABP), expressed in liver and adipose tissue, supports adipogenesis and lipid transport but it is downregulated in laying hens, indicating a shift in lipid metabolism [56, 61]. FABP7 (B-FABP), though more commonly associated with the brain, is expressed in chicken adipose tissues and may be involved in poly unsaturated LCFAs transport [62]. Lastly, FABP10, a liver-specific FABP in birds shows increase expression during the laying phase and plays a role in hepatic lipid transport [56, 63].

### Macro mineral transporters

Minerals, essential for physiological processes, are absorbed primarily in the small intestine, with the duodenum and jejunum serving as key sites for uptake. Minerals can be absorbed via both transporter-mediated mechanisms and passive diffusion. For example, in low calcium (Ca) conditions, transient receptor potential vanilloid 6 (TRPV6) facilitates the apical entry of Ca ions into enterocytes [64], while plasma membrane Ca ATPase (PMCA1b; *ATP2B1*) exports Ca into the bloodstream [65]. Ca can also be absorbed passively through paracellular diffusion when dietary Ca levels are high [66, 67]. The Na/Ca exchanger (NCX1; *SLC8A1*) exchanges intracellular Ca for extracellular Na across the basolateral membrane [68], and calcium-binding protein D28k (Calbindin-D28k; *CALB1*) buffers intracellular Ca, facilitating its transport to basolateral calcium transporters [69]. Na transporters include SGLT1, which co-transports Na with glucose into enterocytes [70], and the epithelial Na channel (ENaC; *SCNN1*), which mediates Na absorption in the distal intestine [71]. Na/K ATPase (Na^+^/K^+^ ATPase; *ATP1A1*) maintains intracellular Na gradients by pumping it out of enterocytes [72]. The NHE3 (*SLC9A3*) exchanges intracellular H ions for extracellular Na [73].

Like Ca, phosphorus (P) absorption in poultry occurs through both active and passive mechanisms, primarily in the small intestine, particularly the duodenum and jejunum. The efficiency of P absorption depends on its form, dietary availability, and intestinal pH. When P concentration in the gut is low, P absorption is primarily mediated by the Na-dependent phosphate transporter protein NaPi-IIb, also known as NPT2b (*SLC34A2*) which is tightly regulated by dietary P levels [74]. Higher P intake downregulates NPT2b mRNA expression, while P deficiency upregulate its gene expression [75, 76]. This process is regulated by the activated form of vitamin D_3_ (1,25-dihydroxycholecalciferol), which enhances NaPi-IIb expression and increases P uptake [77]. The gut also expresses phosphate transporter 1 and 2 (PiT1; *SLC20A1* and PiT2; *SLC20A2*) which are Na dependent [78, 79]. However, when dietary P levels are high, passive paracellular diffusion becomes the dominant route of absorption, allowing P to move between enterocytes down its concentration gradient. This process is not energy-dependent and occurs more efficiently when P is in a soluble inorganic form, as it can freely diffuse across tight junctions [80].

Potassium absorption primarily occurs in the small intestine through both passive and active mechanisms [81]. Passive absorption is facilitated by paracellular diffusion via tight junctions between enterocytes, driven by the electrochemical gradient of luminal K^+^ [82]. Active transport involves the Na^+^/K^+^ ATPase pump located on the basolateral membrane of enterocytes, which maintains intracellular K^+^ levels by exchanging intracellular Na^+^ for extracellular K^+^ [83]. Additionally, K^+^ channels and co-transporters, such as the Na^+^-K^+^-2Cl^−^ co-transporter (NKCC), contribute to K^+^ uptake and regulation within the intestinal epithelium [84]. Dietary factors, including Na intake, can influence the gene expression and activity of these transporters, thereby affecting K^+^ absorption efficiency.

### Trace mineral transporters

Trace minerals in poultry are absorbed through both active and passive mechanisms in the small intestine, particularly the duodenum and jejunum. The absorption route depends on the mineral type, its dietary form, and intestinal conditions. Trace mineral transporters are essential for the absorption and regulation of vital micronutrients, including iron (Fe), zinc (Zn), copper (Cu), manganese (Mn), and selenium (Se).

Fe is mainly absorbed in duodenum in the form of ferrous iron (Fe^2+^) through ZRT/IRT-like protein 14 transporter (ZIP14; *SLC39A14*) and divalent metal transporter 1 (DMT1; *SLC11A2*). ZIP14 is responsible for the initial step of Fe absorption in the intestine, allowing Fe to enter enterocytes, while DMT1 facilitates the transport of divalent metals, including Fe, into the cells [85]. Once inside the enterocytes, Fe^2^^+^ is either stored as ferritin or exported via ferroportin into blood circulation. The hepcidin protein, which is regulated by Fe^2^^+^ levels, further controls the expression of these transporters, ensuring that Fe^2^^+^ absorption is adjusted according to dietary availability [86].

Zn is absorbed in its ionic form of Zn^2+^ in both duodenum and jejunum. The regulation of homeostasis of Zn^2+^ is controlled by a network of ZIP transporters and ZnT (Zn transporters). The ZIP transporters (ZIP1 to 14), encoded by genes in *SLC39A* family, are key transporters involved in Zn absorption in the intestine, allowing Zn to enter enterocytes [87]. On the other hand, ZnT1 (*SLC30A1*) is crucial for exporting Zn from enterocytes into the bloodstream, maintaining intracellular Zn levels [88]. At high Zn^2^^+^ concentrations, passive diffusion through tight junctions increases due to elevated luminal Zn^2^^+^, facilitating movement via paracellular pathways [89].

The duodenum is the major site for Cu absorption, where Cu is primarily absorbed as cuprous ions (Cu^+^). CTR1 (Cu transporter 1, *SLC31A1*) is primarily expressed in the apical membrane of enterocytes and is responsible for transporting Cu^+^ from the intestinal lumen into the enterocytes [90]. Inside the enterocyte, Cu^+^ binds to chaperone proteins for safe intracellular transport before being exported into blood circulation via ATP7A, a Cu-transporting ATPase located in the basolateral membrane of enterocytes [91].

Mn is absorbed in an ionic form (Mn^2^^+^) in both duodenum and jejunum primarily via DMT1 and ZIP8 (*SLC39A8*), both of which contribute to its uptake in the small intestine. DMT1 is only expressed in the duodenum and primarily transports Fe^2^^+^, but it also facilitates Mn^2^^+^ absorption. Since Fe and Mn compete for absorption, low Fe levels increase DMT1 expression, leading to enhanced Mn absorption, whereas high Fe intake can reduce Mn uptake. Mn export to maintain cellular homeostasis is regulated by ZnT10 encoded by *SLC30A10*, a key manganese efflux transporter [92].

Selenium is absorbed throughout the small intestine in the form of selenate (SeO_4_^2−^) or selenite (SeO_3_^2−^) or selenomethionine. SeO_4_^2−^ is absorbed actively via sodium-dependent co-transport, while SeO_3_^2−^ is absorbed passively by diffusion [93]. Selenomethionine, on the other hand, is absorbed actively through amino acid transporters, similar to methionine [93, 94]. Table 1 lists the main minerals and their mechanisms of absorption, transporters, and locations of those transporters.

**Table 1:** Key mineral transporters and their location in the avian small intestine

Mineral	Absorption	Main transporters	Location of transporters
Phosphorus	Active and passive	NaPi-IIb (sodium-phosphate co-transporter); passive diffusion at high luminal P	Apical membrane (brush border) of duodenum and jejunum
Calcium	Active and passive	TRPV6 (apical Ca^2^^+^ channel), Calbindin-D28k, PMCA1b, NCX1; paracellular diffusion	TRPV6: apical, PMCA1b/NCX1: basolateral (duodenum, jejunum); paracellular in all segments
Sodium	Active	SGLT1, NHE3 (apical); Na^+^/K^+^-ATPase (basolateral); ENaC (colon)	Apical membrane (Na^+^ entry); Na^+^/K^+^-ATPase on basolateral membrane
Potassium	Active and passive	Paracellular diffusion (passive), Na^+^/K^+^-ATPase, K^+^ channels, Na^+^-K^+^-2Cl^−^ (NKCC) co-transporter	Mainly paracellular; Na^+^/K^+^-ATPase and K^+^ channels at basolateral membrane
Iron	Primarily active	DMT1, ferroportin,	DMT1: brush border membrane (duodenum); ferroportin: basolateral membrane
Zinc	Primarily active; passive at high Zn levels	ZIP, ZnT1	ZIP4: apical, ZnT1: basolateral (duodenum, jejunum)
Copper	Active	CTR1, ATP7A,	CTR1: apical membrane, ATP7A: basolateral (duodenum)
Manganese	Active	DMT1, ZIP8, ZIP14	Apical membrane (duodenum, jejunum)
Selenium	Active and passive	Selenate: active via Na^+^-dependent sulfate transporter, Selenite (passive), selenomethionine (active via amino acid transporters)	Apical membrane; entire small intestine, varies by selenium species and transporter location

### Other nutrient transporters

In addition to transporters for amino acids and monosaccharides, enterocytes also express transporters that facilitate the absorption of more complex molecules, such as creatine, carnitine, and myo-inositol. Creatine, in its phosphorylated form as phosphocreatine, is crucial for ATP regeneration [95]. The small intestine absorbs dietary creatine through the creatine transporter (CRT; *SLC6A8*) [96]. This process ensures the availability of creatine for cellular energy production, particularly in tissues with high energy demands, such as muscles. Therefore, CRT plays a key role in maintaining energy homeostasis within the body [97].

Carnitine is another nutrient involved in energy production by facilitating the transport of long-chain fatty acids into the mitochondria for oxidation [98]. The organic cation/carnitine transporter 2 (OCTN2; *SLC22A5)*, which mediates carnitine uptake, is expressed in enterocytes of the intestinal villi, as shown by in situ hybridization studies [99]. Further research using immunohistochemistry demonstrated that OCTN2 protein is localized to the brush border membrane of enterocytes, while OCTN3 (*SLC22A21*) is found at the basolateral membrane [99].

Myo-inositol is an essential component of cellular phosphoinositide and plays a critical role in insulin sensitivity, lipid metabolism, and cell survival, structure, and growth [100]. It can be synthesized endogenously from glucose, released from cellular phospholipids, and absorbed from the diet through the intestinal tract [101]. Free myo-inositol is actively transported with high efficiency via three co-transport systems: two sodium-dependent (SMIT1;*SLC5A3* and SMIT2; *SLC5A11*) and one proton-dependent (HMIT; *SLC2A13*) transporter [102].

## Gene expression of nutrient transporters and growth performance in broilers

The expression and regulation of SCL family of transporters which include, as mentioned above, amino acids, peptides, monosaccharides and mineral transporters fundamentally impact the growth of broiler chickens [103, 104]. However, systematic studies correlating the gene expression of nutrient transporters to performance parameters are scanty in the literature and not always consistent. Most studies have conducted the gene expression analysis as an additional set of analysis to the performance without methodical approach to explain the variation in performance by the gene expression. Akram et al. [104] found that birds ranked for high body weight had higher expression of various SLC genes. They specifically reported that the gene expression of EAAT3, rBAT, b^0^^,^^+^AT, y^+^LAT2, and SGLT1 were positively correlated with body weight gain at 7 d of age, whereas there was a negative correlation between expression of some other transporter genes including NaPi-IIb and Na^+^/K^+^-ATPase with body weight gain. Using regression models, they reported that the nutrient transporter ASCT1 amongst 3 other genes related to gut barrier function, immune response and oxidation were highly predictive of the body weight at 7 d of age.

Previous studies have demonstrated a link between gene expression of glucose transporters and higher body weight in broilers [105]. Genes related to glucose efflux, GLUT1 and SGLT1 were highly expressed in high body weight birds [104]. In the same study, upregulation of the fructose transporter GLUT5 in low body weight broilers was reported in a likely adaptive response to counteract nutritional implications of lower feed intake [104]. As shown by Abdel-kafy et al. [106], high body weight birds had higher gene expression of GLUT2 and PepT1 compared with low body weight birds. However, the association of performance with gene expression of nutrient transporters may not always be straightforward. Miska et al. [107] did not find a good correlation between growth potential and gene expression of nutrient transporters but reported that amongst 13 assayed genes, only B^0^AT in the jejunum and EAAT3 in the ileum were correlated with body weight gain. In an earlier study by Miska and Fetterer [108], the expression of monosaccharide transporters GLUT2 and GLUT5 in ileum was generally lower in slow-growing chickens not selected for growth rate, except for GLUT2 in the duodenum and jejunum, compared to modern fast-growing chickens (Ross 308). The authors concluded that the higher gene expression of these genes in Ross chickens suggests that monosaccharide transporters play a key role in rapid growth, and that fast-growing chickens may have an enhanced capacity for monosaccharides absorption. However, in the same study the expression of ASCT1, ATB^0^^+^ and B^0^AT was reported to be higher in slow-growing chickens.

According to Mott et al. [103], the expression of PepT1 and EAAT3 was greater in broiler lines selected for low juvenile body weight compared to those selected for high juvenile body weight. Similarly, in a recent study by Kinstler et al. [109], the mRNA expression of PepT1, particularly on d 3, 5 and 7 post hatch, was upregulated in broiler chickens selected for low body weight (White Plymouth Rocks) compared to similar breed selected for high body weight or fast growing Cobb 500 broilers. The increase in PepT1 mRNA expression in chickens selected for low body weight has been attributed to lower feed intake inducing an anorexic state [109], and has also been observed in chicks subjected to feed restriction [5, 110] and delayed access to feed [111]. Other studies have also demonstrated that growth rate influences the gene expression of nutrient transporters. For example, Zeng et al. [112] compared two Chinese poultry breeds with differing growth rates: the fast-growing and the slow-growing chicks, which requires approximately 105 d to reach a market weight of 1.4 kg. The researchers analyzed intestinal tissue from both embryonic chicks and day-old hatchlings. Among the seven amino acid transporters examined, the transporters that were located at the basolateral side including CAT1, CAT4, y^+^LAT1, and y^+^LAT2, and one transporter located at the brush border side, rBAT, showed significantly higher expression levels in the slower-growing birds. However, CAT2 and b^0,+^AT were not significantly different between the two groups.

In a study by Kalantar et al. [113], despite the upregulation of SGLT1 and GLUT2, birds fed the wheat and barley-based diets had a lower body weight gain and feed efficiency compared to the birds fed corn-based diets. It is possible that the observed upregulation is a natural physiological compensatory response to support performance compared with birds fed corn-based diet.

## Factors affecting the gene expression levels of nutrient transporters

### Age, sex and breed

Age significantly influences the expression of nutrient transporters in the broiler chicken gut, affecting nutrient absorption and overall growth performance. Research studies indicate that the gene expression of nutrient transporters is closely associated with the developmental stage of the intestine [14]. During the late embryonic period, particularly from embryonic d 15 (ED15) to ED21, there is a marked increase in the gene expression of nutrient transporters and digestive enzymes. This upregulation prepares the chick for efficient nutrient absorption post-hatch. For instance, PepT1 and the enzyme aminopeptidase N (APN) show elevated expression levels during this time, facilitating protein digestion and absorption [114]. Immediately after hatching, the small intestine undergoes rapid morphological and functional development. The mRNA expression levels of SGLT1 and GLUT2 in the chicken intestine have been found to be age‐dependent, decreasing from 1 to 3 weeks of age [13]. Li et al. [115] examined the mRNA expression patterns of SGLT1, GLUT2, and GLUT5 in broiler and layer chickens from hatching to d 28 using absolute quantitative real-time PCR. Their results showed that SGLT1 and GLUT2 expression levels increased significantly from d 1 to d 3 before declining until d 28, while GLUT5 expression decreased from d 1 to d 7. The authors concluded that monosaccharide transporter gene expression surges in early intestinal development to meet the high monosaccharide demand for chick growth before d 7, then stabilizes as the intestine matures to maintain nutrient absorption balance. Other studies have also observed age-specific changes in SGLT1 and GLUT5 expression. SGLT1 expression was developmentally regulated, increasing until d 7 before declining by d 14, while GLUT5 expression increased linearly with age [103]. Contrary to monosaccharide transporters, in a comparative study between 1-week-old and 5-week-old broilers, the older group exhibited significantly higher gene expression levels of Na^+^-dependent amino acid transporters, including ASCT1, EAAT3, B^0^AT1, and y^+^LAT1 [116]. However, the authors did not observe any significant differences in the mRNA expression levels of ATB^0^^+^, b^0^^,^^+^AT, rBAT, CAT1, and CAT2. Similarly, in an earlier study, Gilbert et al. [14] reported that the mRNA abundances of PepT1, EAAT3, B^0^AT, SGLT1, GLUT5, and GLUT2 increased linearly with age, while CAT1, CAT2, y^+^LAT1, and LAT1 mRNA levels decreased, suggesting a decline in the expression of latter group of transporters over time. The upregulation of Na^+^-dependent amino acid transporters in older birds corresponds with an enhanced capacity for amino acid absorption in these birds.

The mRNA expression of intestinal Ca and P transporters follows a quadratic pattern with age. CaBP-D28k, PMCA1b, NCX1, PiT1, and NaPi-IIb reach peak expression around 25 d of age, then decline until 40 d of age [117]. Proszkowiec-Weglarz et al. [118] demonstrated that CaBP-D28k mRNA expression is significantly upregulated in both the jejunum and ileum of broiler chickens post-hatch, with peak expression observed at 72 h post-hatch. Following this peak, expression levels decreased and then stabilized in both intestinal segments. The authors also reported a similar expression pattern for the NaPi-IIb transporter gene in both tissues, with the highest mRNA levels detected 4 h post-hatch, followed by a significant decline until 8 d post-hatch, suggesting a transition in P absorption mechanisms as the bird develops. These patterns suggest an initial surge in CaBP-D28k and NaPi-IIb gene expression to enhance Ca and P absorption post-hatch as the chick transitions from yolk sac nutrients to dietary intake. The subsequent decline indicates a reduced mineral absorption capacity in older broilers, likely due to lower Ca and P requirements as skeletal maturation progresses.

Sex-related and breed-specific variations in nutrient transporter gene expression have been reported, primarily due to differences in growth rate and nutrient requirements. Mott et al. [103] found that EAAT3 expression was twice as high in female chicks compared to males. In the same study, PepT1 expression was higher in slow growing breed than in fast growing breed, with peak expression occurring 7 d earlier in females than in males. Specifically, in slow growing birds, peak PepT1 expression occurred at hatch in females and d 7 in males, whereas in fast growing birds, females peaked on d 7, and males on d 14. Overall, fast growing birds exhibited a delayed peak (by 7 d) compared to slow growing birds, regardless of sex. The earlier and higher expression of SGLT1 and EAAT3 in females may indicate a greater need for metabolic efficiency, allowing them to reach the body weight and fat reserves necessary for sexual maturity more effectively [103]. Achieving a positive energy balance is essential for transitioning from growth to reproduction, which involves fat deposition. An enhanced ability to absorb glucose, the primary energy source, and glutamate, the key fuel for enterocytes, at an early stage, may support energy storage and facilitate a positive energy balance sooner.

However, not all studies report the same pattern of nutrient transporter gene expression between males and females. Weintraut et al. [119] found that GLUT2 expression was similar in both sexes in turkeys, while SGLT1 was expressed at higher levels in males. Similarly, Kaminski and Wong [120] studied Aviagen Line A chickens at hatch, d 7, and d 14, reporting that b^0^^,^^+^AT, EAAT3, ASCT1, y^+^LAT2, and GLUT2 were expressed at higher levels in males than in females. The authors also reported a significant sex × age interaction for b^0^^,^^+^AT, PepT1, SGLT1, ASCT1, and y^+^LAT2 mRNA expression, with males exhibiting higher mRNA abundance than females at day of hatch. However, by d 7 and d 14, this difference disappeared, with no significant variation in mRNA expression between males and females in older birds.

Findings on the impact of sex on nutrient transporter gene expression remain inconsistent, making it difficult to determine whether differences in nutrient absorption contribute to performance variations between the sexes. However, a comprehensive understanding of sex-specific nutrient transporter expression could have important implications for poultry nutrition, particularly in diet formulation strategies for both straight-run and sex-separate grow-out operations. The reported early-age difference highlights the need to examine nutrient transporter gene expression in the early post-hatch period. This focus could inform nutritional strategies that address growth rate differences between male and female birds to mitigate inefficient nutrient utilization due to immature intestinal function. These interventions may ultimately enhance performance at market age.

### Site of the gastrointestinal tract

The expression of nutrient transporter genes in the chicken GIT varies significantly across different intestinal segments, reflecting their specialized roles in nutrient digestion and absorption. The duodenum serves as the primary site for mineral absorption, exhibiting high expression of transporters for Ca, P, Zn, Cu, Mn and Fe.

The jejunum, the major site for carbohydrate digestion, has the highest expression of monosaccharide transporters such as SGLT1 and GLUT2, facilitating glucose uptake. The ileum, responsible for peptide and amino acid absorption, shows increased expression of transporters like PepT1 and various amino acid transporters. In alignment with these functional differences, Mott et al. [103] reported that PepT1 and EAAT3 expression was highest in the ileum, moderate in the jejunum, and lowest in the duodenum. Additionally, they found that SGLT1 and GLUT5 expression peaked in the jejunum. Similarly, Gilbert et al. [14] observed that the mRNA abundances of SGLT1, GLUT5, and GLUT2 were highest in the jejunum. In contrast, the ileum exhibited the greatest expression of EAAT3, b^0^^,^^+^AT, rBAT, B^0^AT, LAT1, and CAT2.

Furthermore, Han et al. [121] investigated the spatial expression of Ca and P transporters across different intestinal segments in 21-day-old broilers. Their findings indicated that the duodenum exhibited the highest mRNA levels of CaBP-D28k, NCX1, PMCA1b, and NaPi-IIb, while the ileum showed elevated expression of PiT1 and PiT2 transporters. These findings indicate a declining capacity for active Ca and P absorption from the duodenum to the ileum, with PiT1 and PiT2 playing a crucial role in P absorption in the ileum of broilers. Understanding the segment-specific expression patterns of nutrient transporters can assist in designing poultry diets with targeted nutrient delivery. By formulating diets to match the absorptive capacity of each intestinal segment, nutrient uptake can be maximized, leading to improved growth performance and feed efficiency. For example, incorporating exogenous enzymes, such as phytase, organic minerals, and precision-balanced amino acid profiles, along with a deeper understanding of starch and amino acid digestion dynamics, can further enhance absorption efficiency, ensuring nutrients are utilized where they are most effective.

### Dietary factors

#### Dietary concentrations of the substrate

Dietary substrate concentrations, source and quality affect the expression of nutrient transporter genes in the chicken gut, enabling the intestine to adapt to varying nutrient availability. This adaptive mechanism ensures efficient nutrient absorption and maintains overall metabolic balance. For example, both dietary protein quality and intake levels influence the mRNA expression of nutrient transporters in the gut. Gilbert et al. [5] demonstrated that feed restriction and protein quality significantly influenced the age-related expression of nutrient transporters. In birds on a restricted soybean meal (high quality protein) diet, mRNA levels of PepT1, b^o, +^ AT, EAAT3, y^+^LAT2, and CAT2 increased from d 3 to d 14 of age, while in ad libitum-fed chicks, CAT2 increased less dramatically, PepT1 decreased, and others remained stable. In chicks consuming corn gluten meal (low quality protein), the expression of PepT1, b^0,+^AT, EAAT3, y^+^LAT2, CAT2, and SGLT1 decreased from d 3 to d 7, then increased by d 14. Unlike other transporters, SGLT1 expression remained unchanged with age in soybean meal diets, whether fed ad libitum or restricted, but varied with the corn gluten meal diet. As a low-affinity, high-capacity transporter, PepT1 is a faster and more energy-efficient mechanism for amino acid assimilation compared to free amino acid transporters [26]. Increased PepT1 expression under feed restriction likely represents an adaptive response to conserve energy while optimizing amino acid uptake. This upregulation helps mitigate deficiencies in protein and energy by enhancing the absorption of di- and tripeptides, enabling the bird to maintain essential metabolic functions despite limited dietary resources [5]. Inclusion of 200 g/kg of rice gluten meal in substitution of soybean meal resulted in downregulation of PepT1 and EAAT3 in broiler chickens both at 21 and 42 d of age [122], corroborating by the results of growth performance.

Barekatain et al. [9] reported that feeding broiler chickens a low crude protein diet significantly altered the gene expression of nutrient transporters in the intestine. y^+^LAT1 mRNA was overexpressed in the jejunum of birds fed low crude protein diets compared to standard protein diets and in the ileum compared to high-protein groups, suggesting a compensatory mechanism to enhance amino acid absorption in response to reduced dietary protein levels. In contrast, PepT1 expression in the ileum was downregulated in low-protein diets compared to standard-protein diets but not in high-protein groups, indicating that peptide absorption may be compromised under protein-restricted conditions [9]. Additionally, SGLT1 gene expression in the ileum was upregulated only in birds fed low crude protein diets, likely due to the higher starch concentration in these diets, which increases the demand for glucose absorption. More recently, England et al. [15] showed a significant interaction between dietary crude protein level and sex for the expression of CAT2 and PepT2. These genes were significantly upregulated in females but only when the birds were fed the reduced crude protein diet. Additionally, as a main effect, the reduced crude protein diet upregulated the expression of B^0^AT independent of sex. The authors concluded that broiler chickens can compensate for reduced amino acid availability when fed a low-crude-protein diet by upregulating specific amino acid transporters. Additionally, females appear to adapt better to low crude protein diets than males, likely due to more pronounced upregulation of amino acid transporters [15]. This sex-specific response highlights a potential difference in the physiological mechanisms underlying nutrient assimilation and energy conservation between males and females. Earlier studies also have demonstrated increased expression of certain amino acid transporters in response to feeding a low crude protein diet to broiler chickens [123], indicating a physiological adaptation in order to compensate for the reduction in amino acid substrates. A recent study showed that reducing dietary crude protein by 40 g/kg did not affect the mRNA expression levels of b^0^^,^^+^AT, B^0^AT, CAT1, and CAT2, but downregulated the expression of PepT1 [124]. The observed reduction in PepT1 expression is likely due to a decrease in intact protein levels in the diet. Intact proteins digestion produces peptides that are typically absorbed by PepT1 transporters. As intact protein intake decreases and is replaced by more readily available amino acids, the demand for PepT1-mediated peptide transport diminishes, leading to its downregulation.

Previous research has demonstrated that dietary amino acids can also regulate the gene expression of nutrient transporters in the gut [125–127]. According to Fagundes et al. [128], a methionine-deficient diet impacts not only protein synthesis and feed efficiency but also alters amino acid transporter dynamics. Methionine deficiency upregulated b^0^^,^^+^AT and LAT4 and downregulated LAT1 expressions in the ileum compared to those fed a normal diet. This particularly affects arginine, a cationic amino acid, metabolism, with increased expression of arginine transporters shifting metabolism from nitric oxide to polyamine synthesis. Increased expression of b^0,+^AT in the ileum of the methionine deficient birds could enhance the exchanges of extracellular amino acids [37]. Similarly, Zhang et al. [129] showed that supplementation of diets with L-methionine, DL-methionine, or DL-2-hydroxy-4-(methylthio) butanoic acid influenced the expression of certain intestinal nutrient transporter genes. These alterations were observed as interactions between treatment and bird age, as well as between treatment and specific intestinal segments. The two nutrient transporters most responsive to methionine supplementation were ATB^0^^+^ and B^0^AT. DL-methionine supplementation significantly increased B^0^AT expression in the jejunum and duodenum, as well as ATB^0^^+^ expression on d 5 post-hatch. In contrast, L-methionine supplementation specifically enhanced ATB^0^^+^ expression in the duodenum. As ATB^0^^+^ facilitates the uptake of neutral and cationic amino acids and B^0^AT primarily transports neutral amino acids, their upregulation by DL-methionine supplementation supports increased absorption of not only methionine but also other amino acids. However, the authors did not report changes in expression of PepT1 by the different methionine supplementation, even though expression of methionine transporters was changed [129]. Moreira Filho et al. [130] reported that *in ovo* feeding of threonine increased the mRNA expression of PepT1 in the ileum of broiler chicks at hatch. However, this effect was not observed in chicks at 21 d of age. Dietary tryptophan upregulated the gene expression level of B^0^AT in Chinese broiler breeders [131]. In another study, Jiang et al. [125] investigated the effect of dietary threonine levels in breeder hens’ diet on the gene expression of PepT1 in their offsprings and reported that threonine had no effect on the expression levels of the PepT1 gene in the duodenum or ileum of Chinese yellow-feathered embryonic chicks. The authors argued that the lack of effect on PepT1 gene expression could be attributed to the unchanged threonine percentage and total amino acid composition in the eggs, despite the addition of threonine to the diet of breeder hens of yellow-feathered chickens.

Dietary lysine levels have been reported to affect the gene expression of cationic amino acid transporters [7, 8]. The mRNA expression of CAT1, CAT2 and CAT3 in the liver was upregulated in response to dietary lysine concentration in a dose-dependent manner. Therefore, the authors concluded that alteration of dietary lysine contributes to the gene expression of cationic amino acid transporters, which may alter the growth patterns of broiler chickens through amino acid signalling [7]. Similarly, broiler chicks fed a lysine deficient diet exhibited lower mRNA expression of all CAT isoforms in the liver, *pectoralis* muscle and bursa compared to birds fed diets with adequate lysine. In lysine-deficient birds, *pectoralis* CAT1, CAT2, and CAT3 mRNA expressions were significantly reduced by 437-fold, 126-fold, and 13-fold, respectively, compared to birds fed lysine-adequate diets [132]. Furthermore, the combined expression of high-affinity CAT1 and CAT3 mRNA in chicks on lysine-adequate diets showed a strong correlation (*r*^2^ = 0.51; *P* < 0.001) with tissue growth during lysine deficiency or feed restriction. In addition, dietary lysine-induced alterations to crude protein digestibility have been reported to be accompanied by changes in PepT1, b^0^^,^^+^AT, and CAT1 gene expression [8]. Dietary isoleucine levels were reported to upregulate the mRNA expression of PepT1 in jejunum and LAT1 in ileum of yellow-feathered broilers in a linear or quadratic manner [133]. However, the authors did not report any changes in gene expression of SGLT1 and B^0^AT in response to the dietary level of isoleucine. These results suggest that an optimal dietary level of isoleucine can enhance the expression of intestinal amino acid transporters and protein synthesis-related protein kinase genes, while simultaneously suppressing the expression of proteolytic-related cathepsin genes. In summary, amino acid transporters are differentially expressed throughout the intestine to maintain amino acid homeostasis. As previously discussed, the expression of intestinal nutrient transporter genes varies by intestinal segment and age, highlighting the complexity and dynamism of amino acid absorption. The expression levels of nutrient transporter genes are influenced by both the dietary source and the concentration of amino acids, reflecting the complex and dynamic nature of their regulation.

Hu et al. [76] investigated P absorption and the gene expression of related co-transporters in broilers fed different levels of non-phytate P. Their results showed that increased dietary P at 14 and 21 d inhibited NaPi-IIb mRNA expression in the duodenum and PiT-1 mRNA expression in the ileum, while promoting PiT2 mRNA levels in the duodenum. NaPi-IIb, PiT1, and PiT2 are key transporters for P absorption in the small intestine, with higher P absorption facilitated by upregulating NaPi-IIb and PiT2, while downregulating PiT1. Increased NaPi-IIb expression levels has been reported to be positively correlated to plasma P levels both in the duodenum of broiler chickens [76] and jejunum of laying hens [134]. In another related study, Hu et al. [135] examined the effects of increasing dietary calcium-to-retainable P ratios on Ca and P transporters in the duodenum. Their results showed a linear reduction in the mRNA expression of Ca and P transporters, including CaBP-D28k and PMCA1. Additionally, the expression of NaPi-IIb and PiT2 was either significantly reduced or showed a trend of reduction in a linear manner. These findings suggest that P absorption is not only regulated by the P levels in the feed but also the balance between dietary P and Ca, with high dietary Ca potentially suppressing the expression of key P transporters, thereby influencing P homeostasis in broiler chickens.

Studies have shown that organic or chelated mineral sources with optimal chelation strengths (*Qf*) offer higher bioavailability compared to inorganic sources [136, 137]. Historically, this superiority in bioavailability has been attributed to less dietary interference and antagonism for organic minerals with optimal *Qf*, letting them reach the intestinal brush border where they are hydrolyzed and absorbed as ions, offering higher bioavailability than inorganic forms [138, 139]. However, according to Bai et al. [140] kinetic data suggests that the transport of organic Mn with higher *Qf* is a saturable process, indicating the involvement of at least one distinct transport pathway in the duodenum, separate from the system used for inorganic Mn. These researchers reported that Mn source influenced the expression of DMT1 mRNA, with organic Mn sources showing an increase in DMT1 mRNA expression both in the in situ ligated duodenum and the proximal intestine of the intact broiler model. Organic minerals may also affect the gene expression of other nutrient transporters, rather than their own transporters. For example, supplementing organic chromium (Cr^3^^+^; propionate) in broiler diets has been reported to enhance the expression of nutrient transporter genes in the intestine, with effects varying by intestinal region, transporter type, and age. At 21 d of age, Cr^3^^+^ increased SGLT1 expression in the duodenum, CAT1 expression in the jejunum, and both GLUT2 and CAT1 expression in the ileum. By 42 d of age, Cr^3^^+^ elevated CAT1 expression in the duodenum and enhanced GLUT2 and CAT1 expression in the jejunum and ileum, indicating the potential role of Cr^3^^+^ in modulating nutrient transporter gene expression which resulted in better absorption and utilization of nutrients, thus improved growth rate and feed efficiency [141].

### Fibre, particle size and grain type

The beneficial effects of fibre on the gastrointestinal tract development and nutrient utilization in poultry have been widely recognised. The source, physical structure, and amount of fibre in the diet determines its effectiveness in promoting digestive capacity and nutrient absorption [142]. In this regard, some studies have also investigated the effect of fibre type and source on gene expression of nutrient transporters. Kheravii et al. [6] reported increased expression level of B^0^AT and CAT1 across the small intestine in response to inclusion of sugarcane bagasse as an insoluble fibre source. In addition, coarse particles of corn resulted in upregulation of jejunal ASCT1 and y^+^LAT2, and ileal PepT2. Depending on the overall composition of the diet (i.e., wheat vs. corn based) the gene expression of nutrient transporters may differ. In a study by Gilbert et al. [14], broiler chickens selected on wheat-based diets exhibited higher duodenal PepT1 mRNA expression levels compared to those selected on a corn- and soybean-based diet, indicating a greater capacity for amino acid absorption as peptides in wheat-based diet. However, the study found no significant differences in the mRNA expression levels of 10 amino acid transporters (rBAT, b^0,+^AT, ATB^0^^+^, CAT1, CAT2, LAT1, y^+^LAT1, y^+^LAT2, B^0^AT, and EAAT3), and 4 monosaccharide transporters (SGLT1, SGLT5, GLUT5, and GLUT2) between the two diets. Kalantar et al. [113] showed that birds fed wheat and barley diets had the highest expression of glucose transporters (SGLT1 and GLUT2) and peptide transporter in the jejunum compared with those fed corn-based diets. Tejeda and Kim [143] investigated the impact of fibre inclusion levels and particle size of fibre on jejunal gene expression of SGLT1 and PepT1. While individual factors like fibre type, particle size, and inclusion level did not affect transporter gene expression, the interaction between fibre type and particle size showed that the coarse particle size of cellulose significantly increased PepT1 expression compared to the coarse particle size of soyhulls. However, the poorest growth performance and protein digestibility were for those birds fed diets with coarse particles of cellulose. Similarly, Kheravii et al. [6] observed that broilers fed a 2% sugarcane bagasse diet with a coarse particle size exhibited reduced weight gain alongside upregulation of intestinal CAT1 and PepT2. These findings suggest that the upregulation of nutrient transporters may act as a compensatory mechanism to mitigate reduced nutrient availability caused by lower feed intake. The reduction in feed intake, influenced by fibre content and composition, likely leads to diminished nutrient intake, prompting the upregulation of transporters like PepT1 to enhance nutrient absorption and compensate for dietary deficiencies [143, 144].

### Feed enzymes

Exogenous feed enzymes such as phytase, carbohydrase and proteases are added to the poultry diets to improve nutrient digestibility and utilization by the bird. These enzymes have also been reported to affect nutrient transporter gene expression both in healthy and challenged birds. Exogenous phytase is routinely added to poultry diets to improve P digestibility and mineral utilization [145]. However, phytase has been reported to improve the digestibility of other nutrients such as Na and amino acids [146]. In a study by Walk et al. [147], increasing dietary phytase supplementation in broiler chickens influenced the gene expression of myo-inositol transporters in the small intestine. Specifically, the HMIT exhibited a linear increase in expression in the ileum, while the SMIT2 showed a quadratic increase in the jejunum as phytase supplementation increased. These findings suggest that myo-inositol uptake predominantly occurs in the proximal small intestine via a Na^+^-dependent transporter. Hu et al. [135] reported that phytase inclusion increased serum P levels while upregulating duodenal expression of NaPi-IIb. Specifically, phytase supplementation increased NaPi-IIb mRNA expression by 20% in the duodenum of broiler chickens, whereas the expression levels of another P transporter, PiT2, remained unchanged. Although no research studies currently suggest that phytase supplementation alters the gene expression of amino acid, peptide, or glucose transporters in poultry, studies in swine have demonstrated that phytase supplementation increases the gene expression of PepT1, Ca transporter TRPV6 [148], SGLT1, and GLUT2 [149]. These results could be an indication that phytase may potentially increase peptide and glucose absorption through the upregulation of their transporters in the gut.

Supplementing exogenous multi-carbohydrases to a low energy density diet positively impacted energy and nutrient absorption by enhancing the expression of the intestinal carnitine palmitoyltransferase 1 (CPT1), PepT1 and GLUT2 genes, contributing to improved energy metabolism and growth potential in broiler chickens [150]. Similar upregulation of several intestinal amino acid transporters including B^0^AT, b^0,+^AT, CAT1, CAT2, y^+^LAT1, y^+^LAT2 and PepT1 were observed in response to protease supplementation in broiler chicken diet [151]. The authors also reported linear improvement in body weight gain, feed conversion ratio and protein and energy digestibility in response to dietary protease supplementation. A previous study has established a direct relationship between nutrient digestibility and the expression of intestinal nutrient transporters [148]. For instance, enhanced energy digestibility achieved through supplementation with laminarin, fucoidan, and zinc oxide has been associated with increased gene expression of intestinal glucose transporters [152]. Similarly, Drummond et al. [153] demonstrated that higher availability of essential amino acids stimulates the upregulation of amino acid transporter expression in skeletal muscle. This adaptive response likely serves to enhance intracellular amino acid delivery, supporting improved nutrient utilization and metabolic efficiency.

An interaction between *Eimeria* challenge and enzyme (xylanase and protease) supplementation in birds offered high fibre-adequate protein diets has been reported to result in the upregulation of the expression of GLUT2 and GLUT5 compared to non-challenged birds, potentially compensating for nutrient utilization deficits [12]. Similar xylanase-mediated upregulation of nutrient transporters was observed in broiler chickens challenged with *Clostridium perfringens* [154]. Lin and Olukosi [12] reported downregulation of the expression of amino acid transporters CAT2 and y^+^LAT1 in the jejunum in response to *Eimeria* challenge, while exogenous xylanase and protease supplementation resulted in downregulation of the rBAT transporter.

### Diseases and health challenges

Various disease and health status of the bird influences the expression of genes including that of transporters. The complete discussion of all diseases is beyond the scope of this review and here some of the most common enteric diseases are briefly discussed. Avian coccidiosis, a globally prevalent disease caused by protozoan parasites of the *Eimeria* species, has been shown to alter the expression of various nutrient transporter genes in the gut. The downregulation of nutrient transporter gene expression caused by *Eimeria maxima* and *E. acervulina* challenge has been reported to affect a variety of carbohydrates, amino acid and mineral transporters, such as GLUT2, GLUT5, SGLT1, PepT1, B^0^AT, b^0,+^AT, EAAT3, rBAT, y^+^LAT2 and ZnT1 [32, 155–157]. A study by Miska and Fetterer [158] tracked nutrient transporters mRNA expression levels in response to *Eimeria* infection at the primary lesion sites of duodenum for *E. acervulina*, ileum for *E. maxima*, and ceca for *E. tenella,* and found a common brush-border downregulation of peptide/amino acid transporters including PepT1, B^0^AT, b^0,+^AT, rBAT, EAAT3 and sugar transporters including SGLT1, GLUT2 and GLUT5. Whereas basolateral transporters changed species-specifically where in *E. acervulina* some basolateral transporters including CAT1, y^+^LAT1, y^+^LAT2, LAT1, SNAT2 were upregulated while CAT2 decreased; in *E. maxima* CAT1 and LAT1 showed modest upregulation with small downregulation in others; and in *E. tenella* basolateral transporters were broadly downregulated. Overall, *Eimeria* suppressed apical uptake capacity while driving species-dependent basolateral adjustments. Although the exact causes of this downregulation in apical transporters and changes in basolateral transporters are not fully understood, it is speculated that it serves as a cell-mediated protective response to pathogenic invasion. By reducing the expression of nutrient transporters in epithelial cells, a nutrient-depleted environment is created, which limits parasite development. Additionally, the resulting malnourished cells may trigger apoptosis and subsequent epithelial renewal [157].

However, other researchers have reported downregulation of CAT2 and y^+^LAT2 in response to *Eimeria* infection [12, 159]. CAT2 and y^+^LAT2 are basolateral amino acid transporters mediating the transport of cationic and neutral amino acids [160]. As important L-arginine transporters, the depression of CAT2 and y^+^LAT may contribute to the reported sharp drop of plasma arginine in *Eimeria acervulina* challenged chickens [161]. An infection caused by *Clostridium perfringens* has also led to downregulation of duodenal mRNA expression of SGLT1, PepT1, and L-FABP, while ileal SGLT1 gene expression was increased [162]. In the same study, however, the addition of xylanase upregulated the expression of jejunal SGLT1, PepT1, and L-FABP genes, as well as ileal PepT1 and L-FABP genes in challenged birds. Similarly, Lin et al. [163] found that *Eimeria* challenge downregulated the expression of PepT1, rBAT, CAT2, y^+^LAT2, GLUT2, GLUT5, SGLT1 and zinc transporter ZnT1, while GLUT1 was upregulated.

A decrease in the expression of the basolateral ZnT1 could lead to the toxic accumulation of zinc within epithelial cells, which in turn triggers cell death [32]. The upregulation of GLUT1 may reflect a host strategy to transport more nutrients out of cells, potentially resulting in cellular nutrient depletion and triggering apoptosis [164]. In summary, all the above findings suggest that the growth depression in challenged and unhealthy birds may, at least in part, result from altered expression of digestive enzymes and nutrient transporters in the small intestine, as the cells adjust amino acid transporter expression to reduce the intracellular pool of essential amino acids and glutamate, potentially inhibiting replication of pathogenic agents and triggering cell death. However, the use of exogenous enzymes may help alleviate some of these negative effects in broiler chickens.

The infection with *Campylobacter* species might alter the expression of several genes in the guts of chickens, including nutrient transporters and defensins [165]. Oral infection of *Campylobacter jejuni* reduced the expression levels of SGLT1, GLUT2, PepT1, CAT2, EAAT3 and y^+^LAT2 in different segment of small intestine in broiler chickens at 14 d post infection [166]. In another study, chicks challenged at hatch with 10^6^ colony-forming units *Campylobacter jejuni* upregulated the expression of the Zn transporter ZnT1 at d 7 in the duodenum, ileum and cecum, and the amino acid transporters b^0,+^AT in the duodenum, jejunum and ileum at 14 d [165]. The decreased nutrient absorption, in turn, could also explain the negative effect of a *Campylobacter* spp. colonization on body weight gain and feed efficiency. In addition to the negative effects on growth performance, reduced nutrient uptake by the host may also play a significant role in the persistence of *Campylobacter* spp. colonization. The key steps in *Campylobacter jejuni* colonization involve its adherence to and invasion of intestinal epithelial cells [167]. The bacterium’s ability to acquire essential nutrients is vital for these processes [168]. Conversely, a decrease in the host’s intestinal nutrient absorption could enhance the availability of carbon and nitrogen sources near the mucosal surface, which are crucial for bacterial growth [169]. As a result, changes, whether direct or indirect, in the expression of intestinal nutrient transporters may facilitate the sustained colonization of the gastrointestinal tract and the dissemination of *Campylobacter* spp. to internal organs, as seen with *Campylobacter hepaticus* in spotty liver diseases in layer hens [170].

## Stressors

### Heat stress

Environmental stressors, particularly heat stress, significantly affect the gene expression of nutrient transporters in broiler chickens, disrupting their ability to absorb essential nutrients [11]. Heat stress negatively affects intestinal health in broilers, with its impact depending on both temperature and exposure duration [171]. Reduced feed intake during heat stress can decrease the surface area of the small intestine [172], which in turn affects carbohydrate uptake through glucose transporters [10]. In heat-stressed broilers with significantly lower feed intake, a reduction in GLUT2 expression in the jejunum has been observed, while jejunal SGLT1 expression may remain altered or maintained, suggesting its critical role in glucose absorption under low luminal glucose conditions [10]. However, changes in transporter expression are not solely attributed to reduced feed intake. For example, SGLT1 expression was significantly upregulated in the jejunum of broilers fed ad libitum under heat stress at 30 °C for 14 d, compared to those pair-fed under normal conditions at 20 °C [173]. This indicates that SGLT1 upregulation is a temperature-specific response, independent of feed intake, likely aimed at enhancing glucose absorption to meet the elevated energy demands during heat stress to dissipate heat and keep the body temperature stable (i.e., by panting). This adaptive mechanism helps the body maintain energy homeostasis in high-temperature environments.

**Fig. 3:**
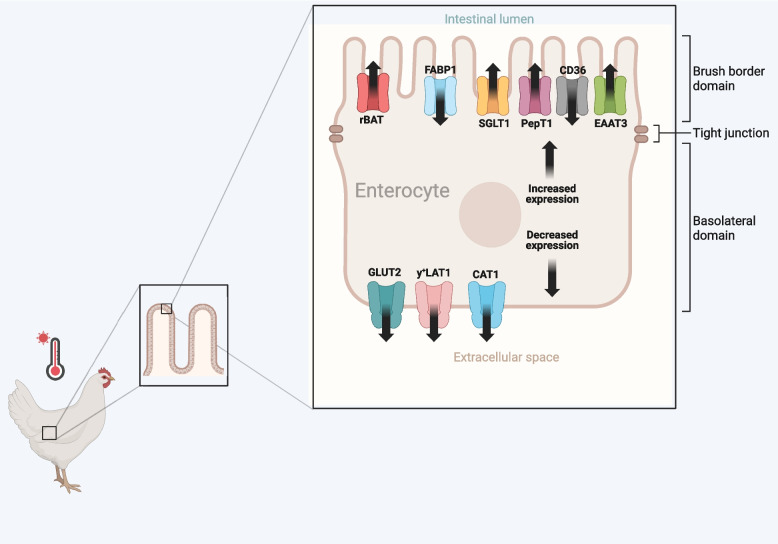
Effects of heat stress on nutrient transporter gene expression in the intestinal epithelium of poultry (created with BioRender.com). This figure shows the differential regulation of key nutrient transporter genes in the enterocytes of heat-stressed broiler chickens. Under elevated ambient temperatures, the expression of several apical (brush border) membrane transporters is upregulated, including SGLT1 (sodium-glucose co-transporter 1), PepT1 (peptide transporter 1), EAAT3 (excitatory amino acid transporter 3), rBAT (related to b^0,+^ amino acid transporter), CD36 (fatty acid translocase), and FABP1 (fatty acid-binding protein 1). These transporters facilitate the uptake of glucose, peptides, glutamate/aspartate, cationic/neutral amino acids, and fatty acids, potentially enhancing nutrient absorption in response to stress-induced metabolic demands. In contrast, heat stress leads to downregulation of basolateral transporters such as GLUT2 (glucose transporter 2), y^+^LAT1 (cationic amino acid transporter 1/2), and CAT1 (cationic amino acid transporter 1), which mediate the export of absorbed nutrients into the bloodstream. This imbalance may impair the efficient delivery of nutrients to peripheral tissues and contribute to compromised growth and productivity

Heat stress has been shown to primarily impact glucose and lipid absorption, while exhibiting a lower impact on amino acid transport. Sun et al. [11] observed that heat stress did not significantly alter the jejunal mRNA expression of key amino acid and glucose transporters such as SGLT1, y^+^LAT1, CAT1, rBAT, and PepT1, but reduced the expression of GLUT2, FABP1, and CD36 (Fig. 3). Similar observations were reported by Al-Zghoul et al. [10], where chronic heat-stress decreased the expression levels of GLUT2 and FABP1 in the jejunum. This suggests that heat stress selectively disrupts glucose and lipid absorption pathways. SGLT1 and GLUT2 play pivotal roles in glucose absorption. While SGLT1 facilitates glucose uptake across the brush-border membrane, GLUT2 mediates transport across the basolateral membrane. GLUT2 can adjust its transport capacity based on luminal glucose concentrations [174], while SGLT1 can transport glucose against its concentration gradient, making it the primary route for glucose absorption when luminal glucose is lower than blood glucose. Habashy et al. [175] observed tissue-specific changes in nutrient transporter gene expression following heat stress. At d 1 post-heat stress, FATP1 and SGLT1 were downregulated in both the *Pectoralis major* muscle and ileum, while FABP1 and PepT1 were exclusively downregulated in the ileum. Interestingly, FABP1 and PepT1 showed opposite expression patterns in the *Pectoralis major*. By d 12, FABP1 was consistently downregulated across both tissues, whereas GLUT1 and PepT2 were specifically downregulated in the ileum, and PepT1 was downregulated in the *Pectoralis major* alone. These findings highlight the nuanced and tissue-specific impact of heat stress on lipid, glucose, and oligopeptide transporters, suggesting potential disruptions in nutrient absorption and retention efficiency. Further elucidating the heat-stress effects on nutrient transporters, Wu et al. [176] reported that heat stress upregulated the relative mRNA expression of SGLT1, CaBP-D28k, and L-FABP in the jejunal mucosa of broiler chickens, indicating an adaptive mechanism to enhance glucose, calcium, and fatty acid absorption under stress.

Excitatory amino acid transporters (EAATs) are a family of glutamate transporters, with EAAT3 being a Na-dependent transporter that has a high affinity for anionic amino acids, particularly aspartate and glutamate [33]. In broiler chickens, EAAT3 gene expression in the jejunum is significantly increased when chicks are exposed to temperatures between 35–39 °C for 1–5 d [177]. Aspartate and glutamate are essential as primary fuels for enterocytes [178], and the increased expression of EAAT3 helps facilitate the uptake of these amino acids, which may play a key role in maintaining intestinal permeability and enterocyte integrity under heat stress [179]. However, it has been noted that the gene expression of aspartate and glutamate transporters is inversely related to growth performance, which may partly explain the decrease in body weight observed in heat-stressed chickens [180]. The discrepancies between studies investigating nutrient transporter gene expression under heat stress can likely be attributed to differences in broiler age and the duration of heat stress exposure. Younger broilers may exhibit more severe disruption in nutrient absorption under heat stress due to their developing intestinal systems, while older birds may have a more stable nutrient absorption capacity. Variations in the type of heat stress (e.g., acute vs. chronic heat stress) and the duration of exposure further influence the extent and type of transporter gene expression.

### Immunological stressors

Other immune stressors, such as inoculation with lipopolysaccharide and cyclophosphamide have been shown to negatively affect bodyweight gain, feed efficiency and simultaneously increase the relative expression of SGLT1, CaBP-D28k and L-FABP mRNAs [181]. These studies suggest that stress-induced physiological changes appear to upregulate nutrient transporter gene expression in broilers. Glucocorticoids released during stress enhance glucose absorption by increasing SGLT1 abundance in the cell membrane [173]. Similarly, CaBP-D28k, a calcium-binding protein, facilitates calcium uptake in the duodenum and jejunum, while FABP1 and L-FABP mediate fatty acid absorption and transport within the small intestine [182, 183]. Stress-related upregulation of CaBP-D28k and L-FABP likely compensates for reduced intestinal absorptive area and meets increased energy demands during stress, highlighting an adaptive response to immune and nutritional challenges. But not all stressors affect the nutrient transporters in the same way. For example, oxidative stress induced by excess dietary lead at 200 mg/kg resulted in downregulation of SGLT1, SGLT4, GLUT2, PepT1 and EAAT3, except GLUT5 mRNA expression which was upregulated [184]. The authors concluded that lead exposure impairs monosaccharide transport in enterocytes by downregulating SGLT1 and SGLT4, which reduces substrates for GLUT2 and leads to decreased GLUT2 mRNA expression. This energy deficit triggers a shift from energy-dependent transporters like SGLT1 and SGLT4 to ATP-independent transport through GLUT5, facilitating passive fructose transport. This compensatory mechanism helps maintain nutrient absorption while conserving cellular energy. These findings are consistent with Douard et al. [185], who observed increased GLUT5 expression under oxidative stress conditions induced by dexamethasone injection, without affecting SGLT1 or GLUT2 expression in rats. Similarly, Barekatain et al. [9] showed that dexamethasone injections significantly affected nutrient transporter gene expression in broilers’ jejunum, where PepT1 and EAAT3 were significantly upregulated in response to dexamethasone injections. However, the expression of b^0,+^AT was unaffected by injections. In another study, Barekatain et al. [186] investigated the gene expression of nutrient transporters in the jejunum and ileum of broiler chickens fed a reduced crude protein diet supplemented with arginine, glutamine, and glycine under dexamethasone-induced stress. The authors observed a significant interaction between dexamethasone treatment and diet for CAT1, where dexamethasone increased the expression of CAT1 only in birds fed additional arginine. Although dietary treatments did not affect the expression of y^+^LAT1, dexamethasone independently downregulated its expression. Similar to the previous studies, increased jejunal expression of SGLT1 in birds injected with dexamethasone was also reported. Activation of SGLT1, has been linked to increased paracellular permeability in the intestine, a phenomenon observed under stress conditions [47] and therefore, the upregulation of SGLT1 expression in the jejunum during stress, supports this connection. Stress-induced SGLT1 upregulation may be a physiological adaptation to meet the heightened demand for water, solutes, and nutrients, suggesting that increased SGLT1 activity during stress could help support nutrient absorption and energy requirements in the face of glucocorticoid-induced metabolic changes.

## Conclusion and implications

This review provides an in-depth examination of nutrient transporters as studied by gene expression and their roles in nutrient absorption, metabolic regulation, and overall birds’ health and performance. Nutrient transporters, primarily from the solute carrier family, mediate the uptake of amino acids, carbohydrates, fatty acids, and minerals. Their regulation is influenced by a combination of dietary factors, developmental stages, environmental conditions, and disease challenges. This dynamic regulation enables the birds to adapt to varying nutritional and physiological demands while maintaining homeostasis and productivity. The expression of nutrient transporters varies across intestinal segments, reflecting site-specific roles in nutrient absorption.

While birds-related factors, like age, sex and site of GIT affect the expression level and pattern of nutrient transporters, external factors such as dietary concentration or source of certain nutrients affect their expression levels and patterns. For example, upregulation of amino acid transporters occurs in response to dietary restrictions or deficiencies, enabling compensatory nutrient uptake. Conversely, low-quality or imbalanced diets can impair transporter dynamics, potentially affecting growth. Given the relationship with the performance parameters and the widespread use of molecular assays in biology, the gene expression of nutrient transporters is a relevant measurement in nutrition and production focused studies to provide insights into the mechanisms by which changes in nutrient digestion, absorption and bioavailability occur. However, it is worth noting that upregulations of nutrient transporter gene expression do not necessarily translate into improved performance such as higher body weight or better feed efficiency, nor does their lower expression is linked to poor performance. Such changes rather let the birds to adapt their physiological responses as a compensatory mechanism to maintain nutrient absorption balance.

Stressors, including heat-stress and infections like *Eimeria* challenge, alter transporter gene expression as an adaptive mechanism. While some downregulate transporters to limit pathogen access to nutrients, others upregulate the gene expression of transporters to meet increased metabolic demands, albeit with negative effect on growth performance. Exogenous enzymes, such as proteases and carbohydrases, demonstrate potential to mitigate these negative effects by enhancing transporter gene expression and improving nutrient digestibility.

In conclusion, changes in nutrient transporter gene expression in response to dietary and environmental factors highlight their critical role in maintaining nutrient homeostasis and resilience in poultry. However, there are obvious limitations in interpretation of mRNA expression results due to lack of robust correlation with protein concentration in general and not only for nutrient transporters. When possible, the protein quantification can help understand the capacity of intestinal nutrient transporters and their functionality in relation to broiler nutrition, growth, health and productivity. The dynamic interplay between gut health, nutrient transporter regulation, and the birds’ adaptation to stress and disease presents significant opportunities for optimizing broiler chicken diets, aiming to improve health and to enhance productivity, potentially reducing the need for antibiotics and improving overall sustainability in meat chicken production.

## Data Availability

Data sharing not applicable to this article as no datasets were generated or analysed during the current study.

## Abbreviations

A-FABP: Adipocyte-type fatty acid binding protein
APN: Aminopeptidase N
ASCT1: Alanine, Serine, Cysteine-preferring transporter 1
ATP7A: Copper-transporting ATPase 1
ATB^0^^+^: Amino acid transporter B^0^^,^^+^
B-FABP: Brain-type fatty acid binding protein
B^0^AT: B^0^-type neutral amino acid transporters
Ca: Calcium
Calbindin-D28k: Calcium-binding protein D28k
CAT: Cationic amino acid transporter
CD36: Cluster of differentiation 36, also known as: fatty acid translocase
CPT1: Carnitine palmitoyltransferase 1
Cr: Chromium
CRT: Creatine transporter
CTR1: Copper transporter 1
Cu: Copper
DMT1: Divalent metal transporter 1
EAAT3: Excitatory amino acid transporter 3
E-FABP: Epidermal-type fatty acid binding protein
ED: Embryonic day
ENaC: Epithelial sodium channel
FATP4: Fatty acid transport protein 4
FABPs: Fatty acid binding proteins
Fe: Iron
GIT: Gastrointestinal tract
GLUT: Glucose transporter
H-FABP: Heart-type fatty acid binding protein
HMIT: Hydrogen-dependent myo-inositol transporter
K: Potassium
L-FABP: Liver-type fatty acid binding protein
LAT1: L-type amino acid transporter 1
LCFAs: Long chain fatty acids
MCT1: Monocarboxylate transporter 1
MLC: Myosin light chains
MLCK: Myosin light chain kinase
Mn: Manganese
NaPi-IIb: Na-dependent phosphate transporter protein
NCX1: Na/Ca exchanger 1
NHE3: Na^+^/H^+^ exchanger 3
NKCC: Sodium-potassium-chloride co-transporter
OCTN2: Organic cation/carnitine transporter 2
P: Phosphorus
PepT1: Peptide transporter 1
PiT1: Phosphate transporter 1
PMCA1b: Plasma membrane Ca ATPase isoform 1b
Qf: Chelation strength
rBAT: Related to b^0^^,^^+^ amino acid transporter
SCFAs: Short chain fatty acids
SGLT1: Sodium-glucose co-transporter 1
SLC: Solute carrier
SMCT1: Sodium-coupled monocarboxylate transporter 1
SMIT1: Sodium-dependent myo-inositol transporter 1
SNAT2: Sodium-coupled neutral amino acid transporter 2
TRPV6: Transient receptor potential vanilloid 6
Se: Selenium
y^+^LAT1: Y^+^L-type amino acid transporter 1
Zn: Zinc
ZnT: Zn transporter
ZIP: ZRT/IRT-like protein transporter
